# Preparation, Characterization, and Oral Bioavailability of Solid Dispersions of *Cryptosporidium parvum* Alternative Oxidase Inhibitors

**DOI:** 10.3390/ijms25137025

**Published:** 2024-06-27

**Authors:** Yongxiang Zhang, Minglang Ma, Jinyu Yang, Xiaotong Qiu, Lin Xin, Yixing Lu, Huiguo Huang, Zhenling Zeng, Dongping Zeng

**Affiliations:** 1 Guangdong Provincial Key Laboratory of Veterinary Pharmaceutics Development and Safety Evaluation, College of Veterinary Medicine, South China Agricultural University, Guangzhou 510642, China; yongxiang@stu.scau.edu.cn (Y.Z.);; 2 National Risk Assessment Laboratory for Antimicrobial Resistance of Animal Original Bacteria, Guangzhou 510642, China

**Keywords:** solid dispersion, hydroxypropyl-β-cyclodextrin, Soluplus^®^, bioavailability, pharmacokinetics

## Abstract

The phenylpyrazole derivative 5-amino-3-[1-cyano-2-(3-phenyl-1*H*-pyrazol-4-yl) vinyl]-1-phenyl-1*H*-pyrazole-4-carbonitrile (LN002), which was screened out through high-throughput molecular docking for the AOX target, exhibits promising efficacy against *Cryptosporidium*. However, its poor water solubility limits its oral bioavailability and therapeutic utility. In this study, solid dispersion agents were prepared by using HP-β-CD and Soluplus^®^ and characterized through differential scanning calorimetry, Fourier transform infrared, powder X-ray diffraction, and scanning electron microscopy. Physical and chemical characterization showed that the crystal morphology of LN002 transformed into an amorphous state, thus forming a solid dispersion of LN002. The solid dispersion prepared with an LN002/HP-β-CD/Soluplus^®^ mass ratio of 1:3:9 (*w*/*w*/*w*) exhibited significantly increased solubility and cumulative dissolution. Meanwhile, LN002 SDs showed good preservation stability under accelerated conditions of 25 °C and 75% relative humidity. The complexation of LN002 with HP-β-CD and Soluplus^®^ significantly improved water solubility, pharmacological properties, absorption, and bioavailability.

## 1. Introduction

*Cryptosporidium* is a protozoan parasite that infects various vertebrate hosts and causes gastroenteritis syndrome [1]. The most common symptom of cryptosporidiosis is watery diarrhea, which may progress to dehydration and shock if untreated [2,3]. Other encountered symptoms may include myalgia, weakness, headache, and anorexia [4]. *Cryptosporidium* infection not only affects human health, but it also seriously threatens the development of animal husbandry [5].

Nitazoxanide demonstrates remarkable efficacy as a broad-spectrum agent against parasitic, bacterial, and fungal infections in animals and humans [6]. It is the sole pharmaceutical approved by the Food and Drug Administration for the treatment of *Cryptosporidium* [7]. However, its use is limited to immunocompetent individuals because it lacks effectiveness in immunodeficient patients [8].

*Cryptosporidium* lacks the tricarboxylic acid cycle and cytochrome-based oxidative phosphorylation pathway typical of traditional energy metabolism and instead primarily relies on an alternate oxidative pathway for oxidative phosphorylation [9]. The alternate oxidation pathway is a branch of the ubiquinone respiratory chain, which is a non-phosphorylated electron transport chain with alternate oxidase (*Cryptosporidium parvum* alternative oxidase, CpAOX) as the terminal oxidase, in mitochondria [10,11]. AOX plays a crucial role in the life cycle of *Cryptosporidium* because this protein is absent in mammals [12]. Given that AOX has become a crucial target for treating *Cryptosporidium* infection, finding and developing CpAOX inhibitors are essential.

Pyrazole, a five-membered heterocyclic ring containing two *ortho* nitrogen atoms, has important biological and pharmaceutical activities in the medical field [13,14]. For example, it is used in the treatment of depression [15] and rheumatism [16] and has antilipemic [17] and antitumor biological functions [18]. Phenylpyrazoles can interact with the γ-aminobutyric acid (GABA) receptors of insects and block the chloride channels controlled by GABA, thus interfering with the normal function of the central nervous system and leading to death [19].

**Figure 1:**
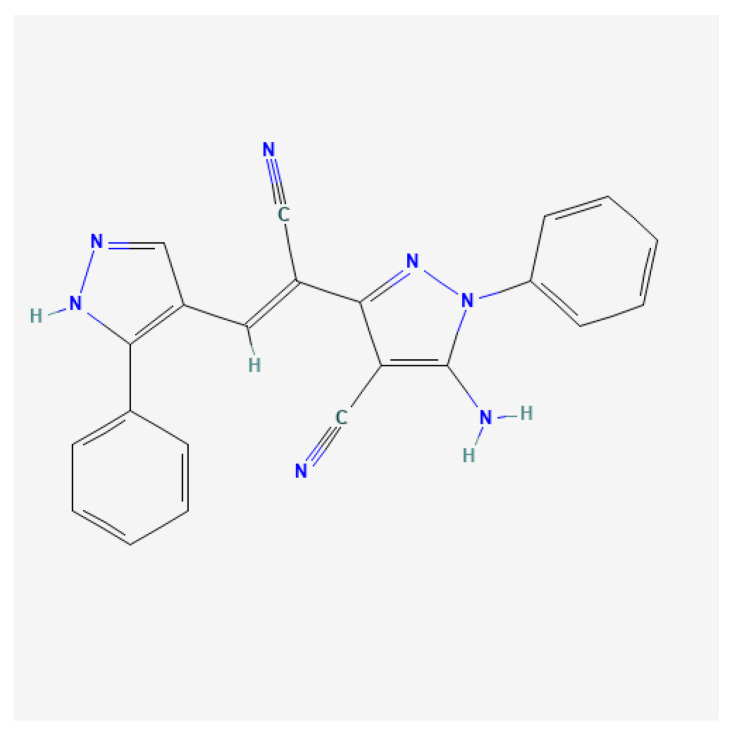
Chemical structure of LN002.

In a preliminary study, dozens of phenylpyrazole derivatives were screened through high-throughput molecular docking, and their anti-*Cryptosporidium* efficacy was studied from multiple angles. 5-amino-3-[1-cyano-2-(3-phenyl-1H-pyrazol-4-yl) vinyl]-1-phenyl-1H-pyrazole-4-carbonitrile (LN002) exhibited promising anti-*Cryptosporidium* activity. Its structural formula is shown in Figure 1. LN002 has low toxicity to mammalian cells, and in rats, its oral LD_50_ exceeds 5000 mg/kg. However, LN002 is almost insoluble in water. Therefore, its oral bioavailability and therapeutic applications are limited. Numerous new preparation techniques and methods have successfully improved the solubility and bioavailability of insoluble drugs. These methods include particle size reduction [20,21], salt formation [22], glycoside modification [23], cyclodextrin inclusion [24], and drug incorporation into solid dispersions or liposomes [25,26]. Among these preparation methods, solid dispersion methods are widely used because they result in excellent solubilization properties [27,28].

In this study, solid dispersions of LN002 were prepared through freeze-drying with HP-β-CD and Soluplus^®^ as carriers. Differential scanning calorimetry (DSC), Fourier transform infrared (FT-IR), powder X-ray diffraction (PXRD), Nuclear magnetic resonance spectroscopy (1H NMR), and scanning electron microscopy (SEM) were utilized to characterize LN002 SDs. The results showed that the solid dispersions were successfully prepared. The saturated solubility, dissolution rate stability, and pharmacokinetics of LN002 and LN002 SDs in rats were evaluated.

## 2. Results Discussion

### 2.1. Preparation and Optimization of LN002 SDs

LN002 SDs were prepared through freeze-drying to enhance solubility, dissolution rate, and bioavailability. The solubility and dissolution rate of hydrophobic drugs in solid dispersions depend on the properties of polymers [29]. The different molecular weights and surface activities of polymers have been found to improve solubility and dissolution rates. In this study, different polymer mass ratios were evaluated to improve the solubility of LN002. In the single-factor experiment, the drug/solid dispersion carrier mass ratios ranged from 1:6 to 1:12, while the solid dispersion carriers HP-β-CD and Soluplus® were added at a fixed mass ratio of 1:1. The single-factor analysis revealed that the optimal drug/solid dispersion carrier mass ratio was 1:12. Similarly to general solid dispersions, solid dispersion solubility increased with the polymer ratio, rather than decreasing [30]. In orthogonal trials, solid dispersions with various effects were prepared by changing the mass ratio of the solid dispersion carrier (HP-β-CD and Soluplus^®^ mass ratios of 1:1, 3:1, and 1:3), reaction temperature (30–50 °C), stirring speed (300–500 rpm), and reaction time (1–4 h). Compared with the solid dispersion prepared with the mass ratio of HP-β-CD to Soluplus ^®^ of 1:1 and 3:1, the solid dispersion prepared with the mass ratio of 1:3 had a large drug load and good solubility. In conclusion, the ratio of drug to polymer is crucial for increasing LN002 solubility. The LN002 SDs with the highest inclusion yield of 89.37% and the highest solubility of 1.124 mg/mL were obtained under the following optimal conditions: HP-β-CD/Soluplus^®^ mass ratio of 1:3, stirring speed of 500 rpm, reaction temperature of 30 °C, and reaction time of 2 h.

### 2.2. Optimal Physicochemical Properties of LN002 SDs

#### 2.2.1. FT-IR

**Figure 2:**
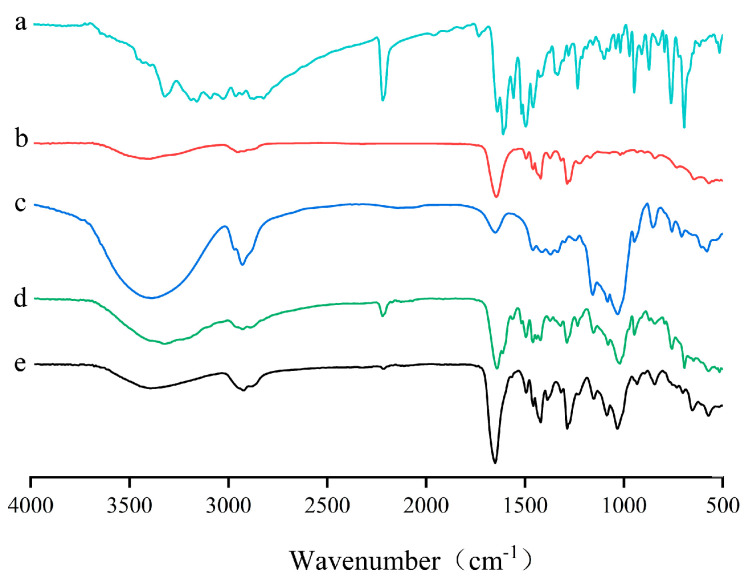
LN002 (**a**), Soluplus^®^ (**b**), HP-β-CD (**c**), LN002 SDs (**d**), and physical mixture (**e**).

The FT-IR spectra of pure LN002, HP-β-CD, Soluplus^®^, LN002 SDs, and the physical mixture are shown in Figure 2. The characteristic absorption peaks of LN002 were found at 3321 (N–H stretching vibration of the primary amine group), 2219 (C≡N stretching), 1640 (N–H bending vibrations), and 762 (N–H bending vibrations) cm^−1^ (Figure 2a). The spectral peaks of Soluplus^®^ were observed at 3397 (O–H stretching), 1645 (H–O–H bending), 1421 (C–H bending vibrations), and 1287 (C–N stretching and NH bending vibrations) cm^−1^ (Figure 2b). The wide absorption band of HP-β-CD at 3000–3700 cm^−1^ was attributed to intermolecular O–H stretching vibrations. Other absorption bands of HP-β-CD appeared at 2930 (C–H bending), 1651 (H–O–H bending), and 1032 (C–O–C stretching vibrations) cm^−1^ (Figure 2c). The FTIR spectra of the physical mixture were similar to the spectra of LN002, HP-β-CD, and Soluplus^®^ monomers, indicating the absence of chemical interactions in the physical mixture (Figure 2d). The absorption peaks of Soluplus^®^ and HP-β-CD moved from 3397 cm^−1^ to 3391 cm^−1^, and the absorption peak of LN002 at 3321 cm^−1^ shifted to 3393 cm^−1^. Wide wavenumber displacement indicates the presence of a hydrogen bond between LN002 and the solid dispersion carrier. The 5-NH_2_ of LN002 is the hydrogen donor, while the secondary OHs of HP-β-CD are a stronger hydrogen bond acceptor than the 5-NH_2_ group. The hydrogen atoms in the NH_2_ group can form hydrogen bonds with the negatively charged oxygen atoms, while the oxygen atoms in the hydroxyl group are partially negatively charged, attracting the hydrogen atoms of the NH_2_ group to form hydrogen bonds [31]. The absorption peaks of the drug in the formulation of LN002 SDs at 3321, 2219, 1640, 762, and 695 cm^−1^ disappeared (Figure 2e). Here, it can be suggested that during the process of encapsulation, LN002 changed from the crystalline state into the amorphous state.

#### 2.2.2. DSC

**Figure 3:**
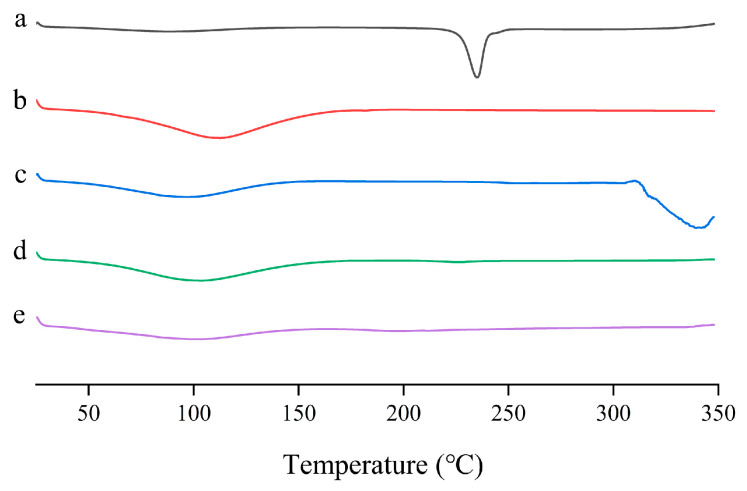
LN002 (**a**), Soluplus^®^ (**b**), HP-β-CD (**c**), physical mixture (**d**), and LN002 SDs (**e**).

The thermograms of pure LN002, HP-β-CD, Soluplus^®^, LN002 SDs, and the physical mixture are displayed in Figure 3. The pure LN002 thermograms showed a separate sharp endothermic peak at 234.99 °C, which corresponded to the melting point of pure LN002 (Figure 3a). Soluplus^®^ did not exhibit any peaks in the range of 25–350 °C (Figure 3b). HP-β-CD presented broad endothermic peaks at 300–350 °C that corresponded to its melting point (Figure 3c). The DSC curves of the physical mixture showed no peaks in the endothermic peak range of 25–350 °C (Figure 3d). The absence of the sharp endothermic peak of LN002 in the thermogram of LN002 SD is a clear indication of the phase transformation of LN002, indicating that the drug was highly dispersed in the solid dispersion, causing the drug to change from a crystalline state into an amorphous state (Figure 3e) [32,33]. Generally, when crystallinity is lower than 2%, the melting peaks of the drug cannot generally be detected with DSC [34]. Therefore, X-ray powder diffraction (PXRD) was used to detect the degree of crystallization of LN002 in solid dispersions.

#### 2.2.3. PXRD

**Figure 4:**
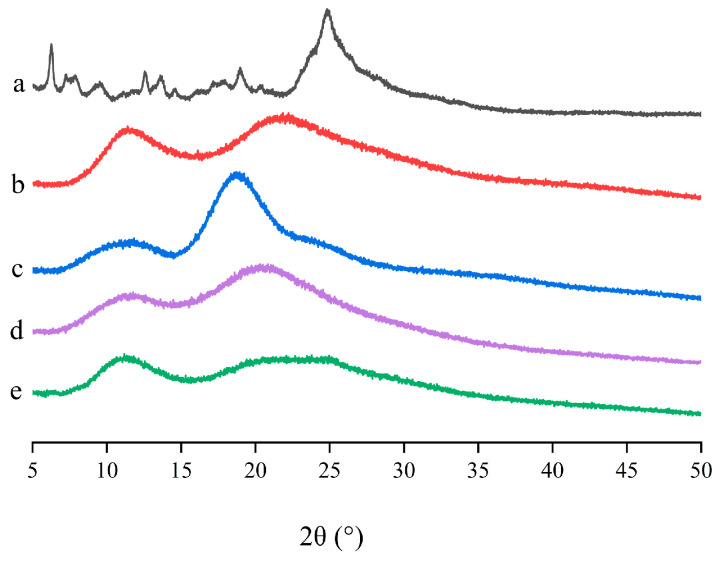
LN002 (**a**), Soluplus^®^ (**b**), HP-β-CD (**c**), LN002 SDs (**d**), and physical mixture (**e**).

The PXRD results of pure LN002, HP-β-CD, Soluplus^®^, LN002 SDs, and the physical mixture are shown in Figure 4. The spectrum of LN002 had characteristic diffraction peaks at diffraction angles (2θ) of 6.26°, 12.55°, 13.59°, 18.97°, and 24.98°, confirming its crystalline nature (Figure 4a). The PXRD spectra of HP-β-CD and Soluplus^®^ lacked crystal peaks, revealing that the two polymer materials were basically amorphous (Figure 4b,c). The PXRD pattern of LN002 SDs exhibited a wide hollow pattern resembling that of Soluplus^®^, and the characteristic peak of raw LN002 could not be observed (Figure 4d), indicating that LN002 transitioned from a crystalline into an amorphous state. The flat PXRD pattern of the physical mixture differed from that of the polymer materials, likely due to grinding (Figure 4e). This result further revealed that the drug was amorphous in the solid dispersion.

#### 2.2.4. SEM

**Figure 5:**
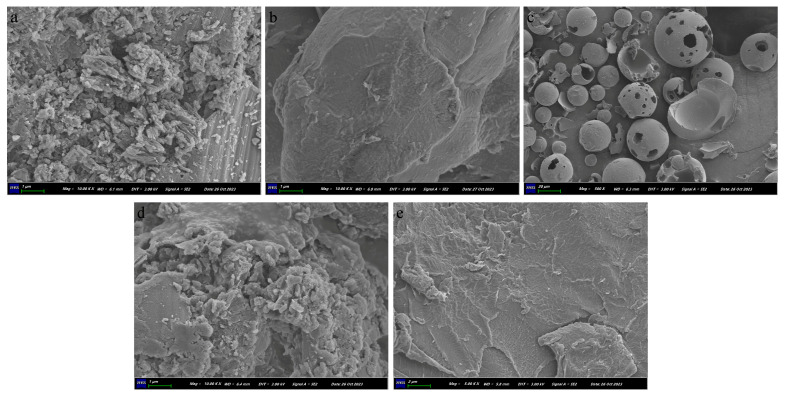
LN002 (**a**), Soluplus^®^ (**b**), HP-β-CD (**c**), physical mixture (**d**), and LN002 SDs (**e**).

The SEM images of pure LN002, Soluplus^®^, HP-β-CD, the physical mixture, and LN002 SDs are displayed in Figure 5. LN002 exhibited irregular granular crystals and compact structures (Figure 5a). Meanwhile, Soluplus^®^ was uniformly dispersed and in an amorphous state (Figure 5b). SEM revealed that HP-β-CD particles presented an amorphous spherical shape with cavities (Figure 5c). The physical mixture exhibited needle-like crystals, indicating that the crystal structure of LN002 did not disappear as a result of physical mixing (Figure 5d). LN002 crystals were not detected in the solid dispersion (Figure 5e). These findings confirmed that LN002 was well encapsulated in the polymer as a result of freeze-drying.

### 2.3. Solubility and In Vitro Release Study

**Figure 6:**
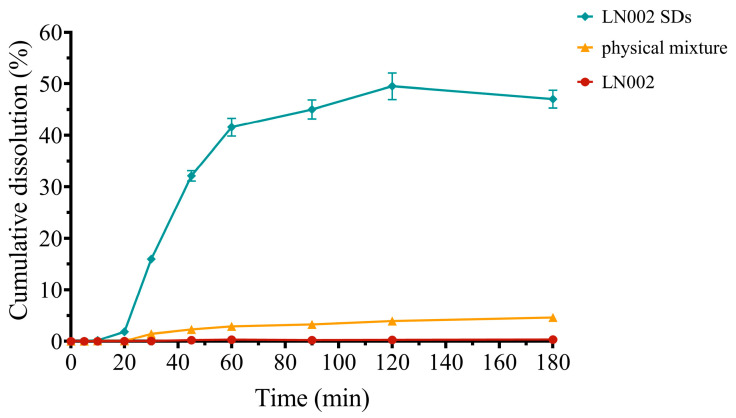
Dissolution curves of LN002, physical mixture, and LN002 SDs.

Saturated solutions of LN002, the physical mixture, and LN002 SDs were prepared and analyzed by using HPLC. Raw LN002 exhibited a low solubility of 0.0236 mg/mL in water. Compared with that of raw LN002, the solubility of LN002 SDs significantly increased to 1.124 mg/mL. The dissolution curves of pure LN002, LN002 SDs, and the physical mixture are provided in Figure 6. The dissolution test results showed that the dissolution rates of LN002, the physical mixture, and LN002 SDs after 180 min were 0.35% ± 0.02%, 4.62% ± 0.21%, and 47.05% ± 1.72%, respectively. Soluplus^®^ is a polymer with micellar properties, which can be used to encapsulate drugs when preparing solid dispersions [35]. In an in vitro release study, the solid dispersing micelle formation and stabilization process reduced the release of LN002 within 20 min. After 20 min, the dissolution rate of the drug increased, which may be due to the stable formation of micelles or increased wettability. These results demonstrated that the solid dispersion significantly improved the dissolution and release of LN002 (*p* < 0.01), indicating that the drug completely transformed from a crystalline state into an amorphous state. This finding was consistent with XRD and DSC results.

In general, solid dispersions can enhance the dissolution of drugs through the following mechanisms. First, with the preparation of the solid dispersion by the hydrophilic carrier, the drug became more wettable and dispersible [36]. In addition, solid dispersions reduced particle size, increasing surface area and dissolution [37,38]. Most importantly, compared with drug powder, solid dispersion can effectively improve the solubility and dissolution rate of water-resistant drugs by adjusting the crystallinity [39].

### 2.4. Stability Study

**Figure 7:**
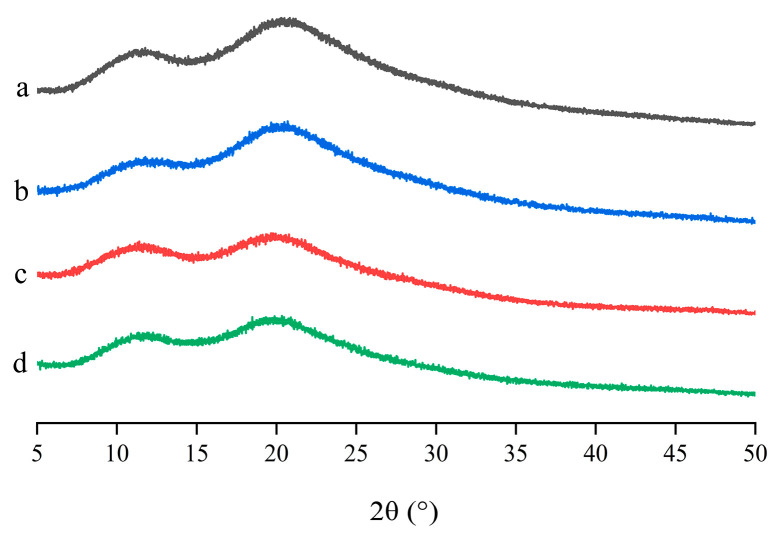
PXRD spectra of LN002 SDs stored for 0 (**a**), 30 (**b**), 60 (**c**), and 90 (**d**) days.

The stability study results of LN002 SDs are provided in Figure 7. PXRD data showed that LN002 SDs remained amorphous within 90 days likely because the solid dispersion can provide physical or steric hindrance. It is well known that Soluplus^®^ can reduce supersaturation by increasing the equilibrium solubility of drugs, thereby achieving the effect of inhibiting crystallization [40,41]. Soluplus^®^ and HP-β-CD, as carriers of the solid dispersion, facilitated the preservation of LN002 in its amorphous state for a long time, which is important to inhibit the crystallization of poorly soluble drugs in the amorphous state.

### 2.5. In Vivo Pharmacokinetics Study

**Figure 8:**
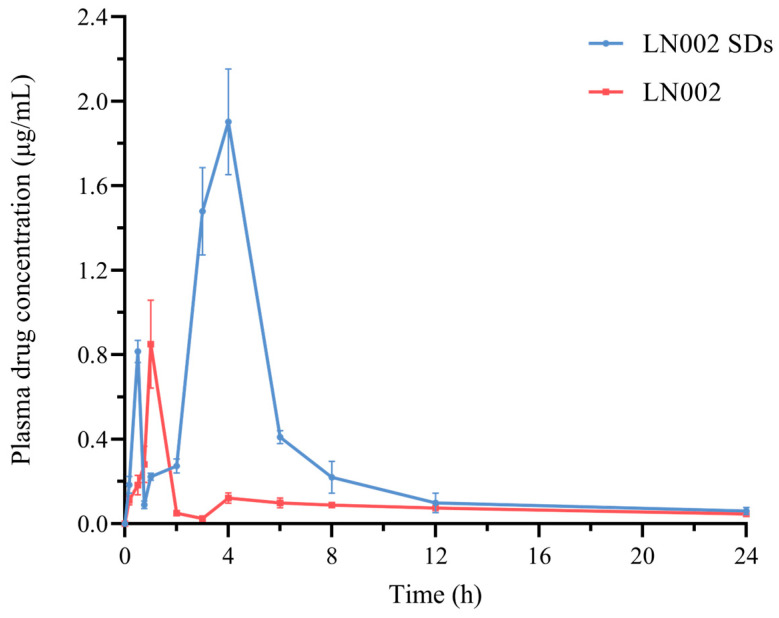
Mean blood concentration–time curve of LN002 SDs and LN002 after oral administration (mean ± SD, n = 6).

The mean blood concentration–time curves and main pharmacokinetic parameters of LN002 and LN002 SDs in rats are provided in Figure 8 and Table 1. At almost all time points, the plasma drug concentration of LN002 in LN002 SDs was higher than that of raw LN002, indicating that the solid dispersion agent could effectively increase the plasma drug concentration of LN002. The drug–time curves of LN002 SDs and pure LN002 showed that the oral administration of the drug involved two distinct absorption processes. The first absorption time of LN002 SDs occurred 0–0.5 h after administration, and that of LN002 occurred 0–1 h after administration, which is the conventional absorption process in oral administration. The second absorption period occurred 1–4 and 3–4 h after administration. The mean blood concentration–time curves exhibited double peaks due to enterohepatic circulation, delayed gastric emptying, or reabsorption in various parts of the gastrointestinal tract [42,43]. Compared with that of the LN002 suspension, the oral administration of LN002 SDs showed significantly increased Cmax and AUC0-t values. Statistical analysis revealed that the Cmax values of LN002 SDs and LN002 were 1.97 ± 0.11 and 0.85 ± 0.19 μg/mL, respectively, and the Cmax of SDs was 2.32 times that of the LN002 suspension (*p* < 0.01). Specifically, the AUC0-t of LN002 released from LN002 SDs (7.72 ± 0.50 μg/mL·h) increased by 3.38-fold compared with that of the LN002 suspension (2.28 ± 0.23 μg/mL·h) (*p* < 0.01). Cmax and AUC in LN002 SDs were higher than LN002, which is consistent with the results of dissolution in vitro. The improved bioavailability of LN002 can be attributed to its increased solubility when incorporated into the solid dispersion carrier, thereby facilitating drug dissolution and absorption in the gastrointestinal tract [44,45].

## 3. Materials and Methods

### 3.1. Materials and Instruments

Raw drug samples were provided byRuipu Biotechnology Co., Ltd. (Tianjin, China). DMF was purchased from Damao Chemical Reagent Factory (Tianjin, China). HP-β-CD was obtained from Maclin Biochemical Co., Ltd. (Shanghai, China lot: 12087655). Soluplus^®^ was purchased from BASF, Ludwigshafen, Germany. A Shimadzu LC-20A system (Shimadzu Technologies, Kyoto, Japan) equipped with a LabSolutions system (version 5.1) system was used for high-performance liquid chromatographic (HPLC) analysis. Chromatographic-grade methanol was purchased from Thermo Fisher Technology (Shanghai, China). The separation process utilized an ARD-C18 column (250 mm × 4.6 mm, 5 µm) purchased from Zhongpu Technology Co., Ltd. (Fuzhou, China). 

### 3.2. Solid Dispersion Preparation

LN002 solid dispersions were prepared through freeze-drying. Briefly, raw LN002 and the solid dispersion material (including HP-β-CD and Soluplus^®^) were co-dissolved in 8 mL of DMF at a ratio of 1:6, 1:9, or 1:12 (*w*/*w*). The HP-CD/Soluplus^®^ (*w*/*w*) ratios were 1:1, 1:3, or 3:1. The mixed DMF solution was stirred at a certain speed for several hours and stored in a refrigerator at −80 °C. The frozen samples were freeze-dried to obtain solid dispersions. A physical mixture consisting of LN002 mixed with Soluplus^®^ and HP-β-CD was prepared.

### 3.3. Complex Characterization

#### 3.3.1. FT-IR Spectroscopy

Each sample was homogeneously mixed with KBr powder at a mass ratio of 1:100 and pressed. Raw LN002, HP-β-CD, Soluplus^®^, the physical mixture, and LN002 SDs were scanned by a Nicolet iS50 FT-IR spectrometer (Thermo Fisher Scientific, Waltham, MA, USA). FT-IR spectra were scanned within the wavenumber range of 4000–400 cm^−1^. Lastly, the resulting image was created using Origin 2021 (OriginLab Corporation, Northampton, MA, USA).

#### 3.3.2. DSC

The differential scanning calorimeter TA-Q20 (TA Instruments, New Castle, DE, USA) was used to monitor thermal behavior to verify the existence state of the prepared samples. The heating rate was set at 10 °C/min, nitrogen was used as the carrier gas, and the temperature range was 25–350 °C. The Origin 2021 software (OriginLab Corporation, Northampton, MA, USA) was used to arrange the data.

#### 3.3.3. X-ray Powder Diffraction

The X-ray diffraction (XRD) data for each sample were acquired by using a SmartLab diffractometer (Rigaku, Japan) at 40 kV and 40 mA with Cu Kα radiation (λ = 1.54056 Å). The 2θ scanning range was set from 5° to 50° with a step size of 0.01° and calculation time of 1 s per step. The outcomes were shown using a program Origin 2021 (OriginLab Corporation, Northampton, MA, USA).

#### 3.3.4. SEM

The surface morphologies of LN002, HP-β-CD, Soluplus^®^, LN002 SDs, and the physical mixture were acquired by using a Zeiss Sigma 300 scanning electron microscope (Carl Zeiss, Oberkochen, Germany) equipped with image acquisition software (version 7.0.5). The samples were affixed to an aluminum sample holder and coated with a layer of gold. Subsequently, the samples were observed under the scanning electron microscope at an acceleration voltage of 10 kV.

### 3.4. Saturation Solubility Analysis

Excess amounts of LN002 and LN002 SDs were added to distilled water. All samples were shaken for 48 h at 37 °C in a thermostat oscillator (Sunkun, China). After 15 min of centrifugation, the supernatants were collected and filtered through a 0.22 μm membrane filter. After appropriate dilutions, the dilutions were analyzed by HPLC. The analytes were identified utilizing a UV detector at 333 nm. Chromatographic conditions used an isocratic mobile phase that combines methanol (solvent A) and deionized water (solvent B) in a ratio of 75:25 (*v*/*v*), with a constant flow rate of 1 mL/min to ensure consistent separation. The injection volume of 20 μL was utilized, and the column temperature was maintained at 40 °C. Experiments were conducted in triplicate to minimize deviations.

### 3.5. In Vitro Dissolution Studies

The in vitro dissolution kinetics experiment on LN002, the physical mixture, and LN002 SDs was performed with a USP type II dissolution apparatus. In brief, pure LN002 (100 mg), LN002 SDs (containing 100 mg of LN002), and the physical mixture (containing 100 mg of LN002) were accurately weighed and placed in 200 mL of phosphate buffer (PBS pH 6.8). In accordance with the published protocols, the PBS was prepared [46]. During dissolution, the dissolution medium temperature was maintained at 37 °C ± 0.5 °C, and the stirring speed was controlled at 100 rpm. At predetermined time points (5, 10, 20, 30, 45, 60, 90, 120, and 180 min), exactly measured samples of the dissolution medium were removed and replaced with the same volume of prewarmed fresh dissolution medium. The collected solution was filtered through a 0.22 μm membrane filter, appropriately diluted, and then detected at 333 nm with an LC-20A UV detector (Shimadzu, Japan). All experiments were performed in triplicate, and the standard regression curve equation was used to calculate the dissolution rate.

### 3.6. Stability Study

LN002 SD samples were maintained in a controlled environment at a constant temperature of 25 °C ± 0.5 °C and relative humidity of 75% ± 5% for 90 days. PXRD analysis was performed at time points of 0, 30, 60, and 90 days to monitor the potential crystallization of the solid dispersion.

### 3.7. In Vivo Pharmacokinetic Studies

Two groups of 12 rats (*n* = 6) were randomly assigned for pharmacokinetic studies [47]. Rats (200 ± 20 g) were fasted for 12 h before the experiment with access to water. The rats in the LN002 and LN002 SD groups were intragastrically administered LN002 or LN002 SDs at a dosage of 100 mg/kg. Blood samples were collected from the tail vein at 0.17, 0.5, 0.75, 1, 2, 3, 4, 6, 8, 12, and 24 h after administration. The collected blood samples were centrifuged at 3000 rpm for 10 min at 4 °C to isolate plasma. Subsequently, 0.2 mL of plasma was mixed with 0.8 mL of acetonitrile, vortex-mixed, and centrifuged at 12,000 rpm for 10 min. The resulting supernatants were filtered by using a 0.22 μm membrane filter and subjected to analysis. Plasma pharmacokinetic parameters in single-dose studies were evaluated by using noncompartmental analysis in Phoenix WinNonlin^®^ 8.2 software (Certara, L.P., Princeton, NJ, USA).

## 4. Conclusions

LN002 SDs, which were prepared by using an optimized composition of HP-β-CD and Soluplus^®^, significantly enhanced the solubility of LN002 in water by 47.50–52.13 times relative to that of LN002 alone. DSC, FTIR, SEM, and PXRD revealed that in SDs, the drug existed in an amorphous state, indicating that LN002 stably dispersed in solid dispersions. Furthermore, the pharmacokinetic analysis of LN002 SDs in rats demonstrated that the pharmaceutical properties of the absorption (C_max_: 0.85 μg/mL vs. 1.97 μg/mL) and bioavailability (2.28 μg·h/mL vs. 7.72 μg·h/mL) of LN002 significantly improved. The results showed that LN002 SDs may serve as a promising drug delivery system for enhancing the solubility and bioavailability of LN002.

## Figures and Tables

**Table 1:** Pharmaceutical parameters of LN002 and its solid dispersions.

Parameter	Unit	Value	
		LN002	LN002 SDs
*T_max_*	h	1	3.83 ± 0.41
*C_max_*	μg/mL	0.85 ± 0.19	1.97 ± 0.11 **
*T*_1/2_	h	18.03 ± 5.82	8.45 ± 1.37
AUC_0–t_	μg·h/mL	2.28 ± 0.23	7.72 ± 0.50 **

** *p* < 0.01, compared to LN002.

## Data Availability

The corresponding authors will make the data supporting this study available upon reasonable request.
